# Persistence of avian carcasses on sandy beaches and marsh edges in the northern Gulf of Mexico

**DOI:** 10.1007/s10661-019-7920-3

**Published:** 2020-03-17

**Authors:** Veronica W. Varela, Guthrie S. Zimmerman

**Affiliations:** 1 Natural Resource Damage Assessment and Restoration Program, U.S. Fish and Wildlife Service, 1011 E Tudor Rd, MS 361, Anchorage, AK 99503 USA; 2 Division of Migratory Bird Management, U.S. Fish and Wildlife Service, Sacramento, CA 95819 USA

**Keywords:** Avian carcass, Persistence, Beach, Marsh, Gulf of Mexico, *Deepwater Horizon*

## Abstract

As part of the natural resource damage assessment for the *Deepwater Horizon* oil spill, a mathematical model was used to estimate the total number of bird carcasses deposited on shorelines based on the number of carcasses collected and adjustment factors such as detection probability and carcass persistence. Studies of carcass persistence occurred along sandy beaches and marsh edges in the northern Gulf of Mexico to obtain site-specific inputs for the model. We estimated persistence rates for these habitat types and evaluated the influence on persistence of carcass size, location of the carcass on the beach, dominant vegetation type in the marsh, carcass distance into marsh vegetation, and length of time a carcass was stranded on a shoreline. The length of time stranded had the greatest influence on persistence in both habitat types, with persistence initially relatively low and increasing logarithmically. Carcass size and position were weakly influential on sandy beaches. Carcass size had stronger influences along marsh edges, and marsh habitat type also affected persistence. We found evidence of a positive relationship between distance into the marsh and persistence during the first 24 h after carcass deployment.

**Electronic supplementary material:**

The online version of this article (10.1007/s10661-019-7920-3) contains supplementary material, which is available to authorized users.

## Introduction

During an oil spill in coastal environments, the number of bird carcasses found on shorelines can be used as an indicator of the magnitude of environmental harm. However, the number of carcasses found on a shoreline is typically less than the true number that was deposited on the shoreline, because the efficiency of searchers is rarely perfect (detection probability) and carcasses may be removed from shorelines by animal scavengers or other actions (carcass persistence). A mathematical model is commonly used during oil spill natural resource damage assessments to estimate the total number of bird carcasses that deposit on shorelines based on the number of carcasses collected and adjustment factors for at least detection probability and carcass persistence (Ford et al. 1996, 2001, 2006, 2009).

The *Deepwater Horizon* (DWH) oil spill began on April 20, 2010 when an explosion and fire occurred on the *Deepwater Horizon* oil rig while drilling a well at the Macondo Prospect (Mississippi Canyon Block 252) in the northern Gulf of Mexico. Approximately 134 million gallons of oil escaped the uncontrolled well over 87 days, eventually impacting shorelines from Texas to Florida (*Deepwater Horizon* Natural Resource Damage Assessment Trustees 2016). Authorized state and federal governmental agencies formed a Natural Resource Damage Assessment and Restoration Trustee Council (“Trustees”) to conduct a comprehensive natural resource damage assessment (NRDA) to quantify the harm done by the oil spill to natural resources and to identify necessary restoration activities. As part of the process to quantify harm to birds, the Trustees counted and collected carcasses from shorelines in the vicinity of the oil spill and conducted several field studies to develop inputs for a mathematical model that would estimate total bird carcass deposition on shorelines (Amend et al. 2020). To estimate the carcass persistence inputs for the model, the Trustees evaluated how long deployed study carcasses remained on shorelines of sandy beaches and marsh edges in the northern Gulf of Mexico. The investigators focused on developing carcass persistence estimates for different shoreline types (i.e., sandy beaches vs marsh edges, which may indicate different types of scavengers or other carcass removal activities) as a function of time and carcass size.

Other factors may influence avian carcass persistence, such as location of the carcass on the shoreline relative to the surf, distance into marsh vegetation from the vegetation/open water interface, and the age of the carcass (i.e., amount of time the carcass has been dead and decomposing). Human interference, such as an individual depositing a carcass into a trash receptacle or the large-scale mechanical trash removal and grooming of beaches for esthetic purposes, can also affect the persistence of carcasses in recreational areas. The field studies were not intended to quantify the specific influences of these other potential factors on carcass persistence; however, investigators recorded information relating to these factors during the field studies. Using the available data, we examined the potential influence of these factors on carcass persistence on sandy beaches and marsh edges in the northern Gulf of Mexico to provide information for consideration by future investigators of carcass persistence.

## Methods

### Study area

The NRDA field studies focused on the two habitat types that comprised the majority of shorelines in the northern Gulf of Mexico from the Texas-Louisiana border to Cape San Blas, Florida: sandy beaches and estuarine marshes. Sandy beaches were fine-grained sediment firm enough to support searchers walking along the beach looking for bird carcasses. During the spill, searches spanned the width of a sandy beach between the water’s edge and the start of terrestrial vegetation; this zone comprised the study area for the sandy beach carcass persistence study. The estuarine marshes were comprised primarily of *Spartina* sp. or *Phragmites* sp. emergent vegetation and the NRDA field study focused on the “edges” of these marshes. The “marsh edge” was a zone of transition between open water and emergent vegetation that varied in width. At lower tides, the “edge” was sometimes identified by vertical ledges of peat, but usually the transition between the open water and marsh edges were not hard boundaries. Often marsh edges did not have a firm substrate upon which a searcher could stand without causing damage to the marsh, so searches for bird carcasses along marsh edges during the spill occurred from boats.

### Study carcasses

No birds were killed for purposes of implementing either of the carcass persistence studies. Most of the study carcasses were salvaged from various legally permitted animal control programs throughout the USA that collected birds using firearms with non-toxic shot or by live capture and euthanasia. A minority of the carcasses were provided by natural resource management agencies that had collected specimens in good condition through routine management activities. None of the study carcasses were affiliated with oil spills or disease-related mortality events. Birds were stored frozen until prepared for use in the studies.

The species and sizes of birds available for use in the field studies were limited to those targeted by the carcass sources described above, and this constrained the flexibility of the field investigators in designing the distribution of study carcasses particularly within size categories. Four sizes of carcasses were used: small (< 200 g), medium (200–500 g), large (501–1000 g), and extra-large (> 1000 g). Examples of the spill-impacted birds in the four size categories include sanderling (*Calidris alba*), laughing gull (*Leucophaeus atricilla*), great egret (*Ardea alba*), and brown pelican (*Pelecanus occidentalis*), respectively. Confounded by the relative unavailability of small and extra-large carcasses from carcass suppliers for use in the studies, the field investigators designed the size distributions of study carcasses to mimic the distributions that were found in the carcasses that were collected during the spill. Prioritizing equal sample sizes among carcass size classes would have resulted in very small sample sizes overall. The study carcasses used were representative of the species known to be affected by the DWH incident. More than 8700 birds from 149 species were collected dead or impaired in the incident area during wildlife rescue and NRDA operations, excluding birds that likely died of other causes. Of these, 57% were species from the family Laridae of relatively similar size (medium) and appearance: 45% were gulls (subfamily Larinae), with an additional 12% consisting of terns (subfamily Sterninae) and black skimmers (*Rynchops niger*). For the sandy beach and marsh edge persistence studies, respectively, 58% and 59% of the study carcasses were species from the family Laridae.

### Sandy beach field study

**Fig. 1:**
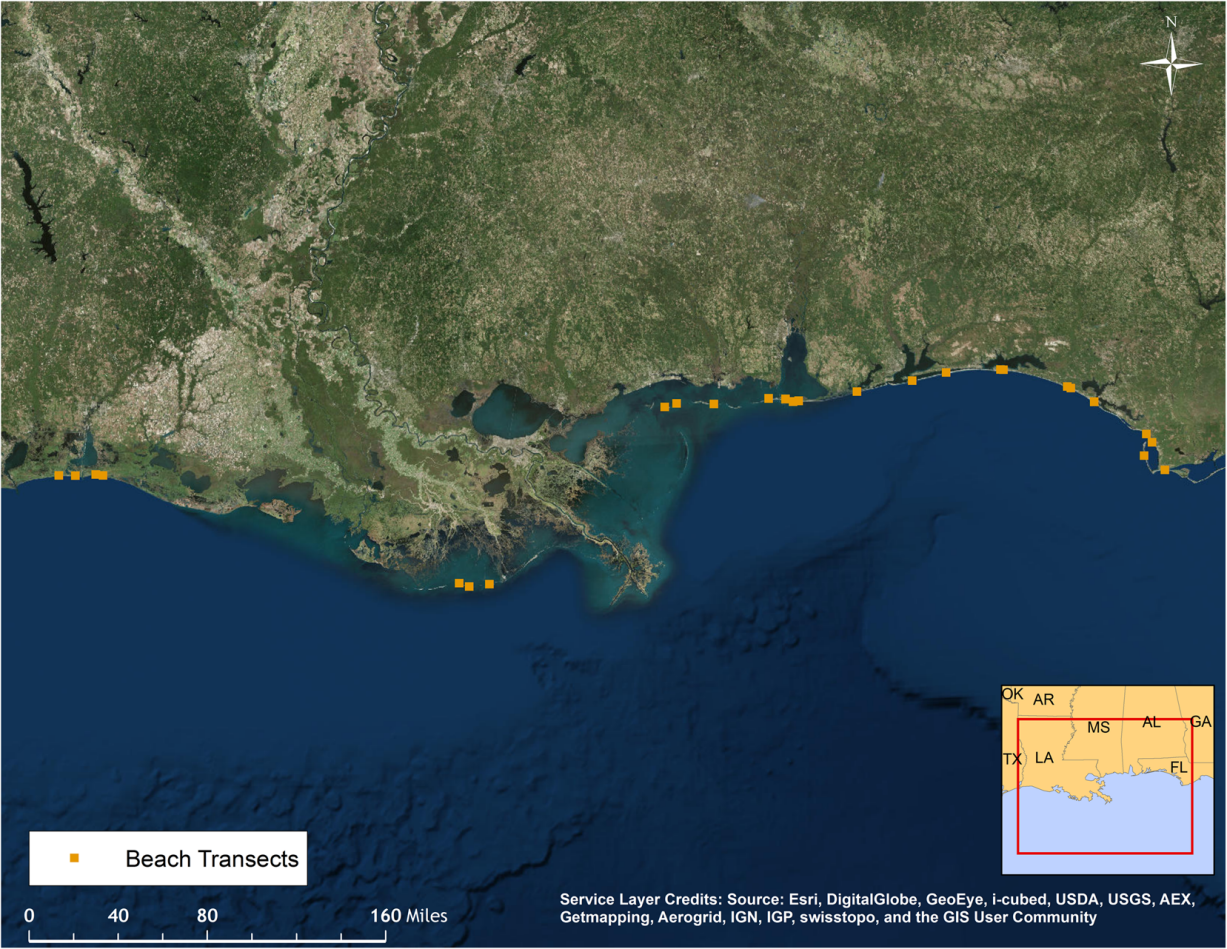
Locations of sandy beach study transects within the study area for the carcass persistence study

Carcass persistence on sandy, walkable beaches was evaluated in late June 2011 (Donlan et al. 2014). Each study carcass was uniquely marked with numbered poultry tags. One hundred thirteen non-oiled, intact bird carcasses were placed in random, predetermined locations on 26 transects along sandy beaches in the northern Gulf of Mexico between the Texas-Louisiana border and Cape San Blas, Florida (Fig. 1). The sandy beaches targeted for persistence study transects were located in the zone of the spill area from which the vast majority of dead and moribund birds were collected as a result of the DWH incident. This zone was also the zone to be used by the Trustees to model total acute avian mortality. The sandy beach transects chosen were roughly evenly distributed geographically throughout this zone to the extent logistically possible, as the objective of the study was to provide specific model inputs that were applicable to the entire modeled sandy beach area. Placement of each carcass on the beach was directed to one of three zones: lower beach (below/seaward of the high tideline; a.k.a., intertidal zone or wash zone), wrack (area of debris deposited at high tide line), and upper beach (landward of the high tide line, but seaward of upland vegetation). Four sizes of carcasses were used, but large and extra-large carcasses were grouped into a single “large” category for field implementation. A wooden block was placed under each deployed carcass as a tool to decipher the fate of a carcass that moves or disappears. If the block remained in the carcass’s last known location but the carcass was missing, removal by scavengers was suspected as the cause of disappearance. If the carcass and block were both missing, tidal action or human intervention were suspected as the cause of disappearance. These observations assisted field researchers trying to verify the disappearance of a carcass.

Each carcass was relocated using GPS every day after deployment for 14 days, weather permitting, or until the carcass disappeared, whichever occurred sooner. Key to interpreting whether a carcass persisted from day to day was whether the carcass remained in the zone of the beach that would have been searched by beached bird survey teams during the NRDA surveys in 2010 (from water line, through wrack zone, to beginning of upland vegetation or dune vegetation, as relevant) regardless of whether it remained in its exact deployment location. If a carcass appeared to be absent from its last known GPS location, field teams searched a larger area of the beach to verify the disappearance of the carcass. Carcasses found outside of the NRDA search area or found totally buried within the NRDA search area were considered “missing.” Carcasses considered to be remaining on the beach must have been in carcass condition states consistent with the definition of a “bird carcass” provided by the beached bird survey protocols used by spill responders in 2010: “A bird carcass is defined as any dead bird, regardless of its condition. As little as a few feathers attached to skin fragments constitutes a bird carcass.”

### Marsh edge field study

**Fig. 2:**
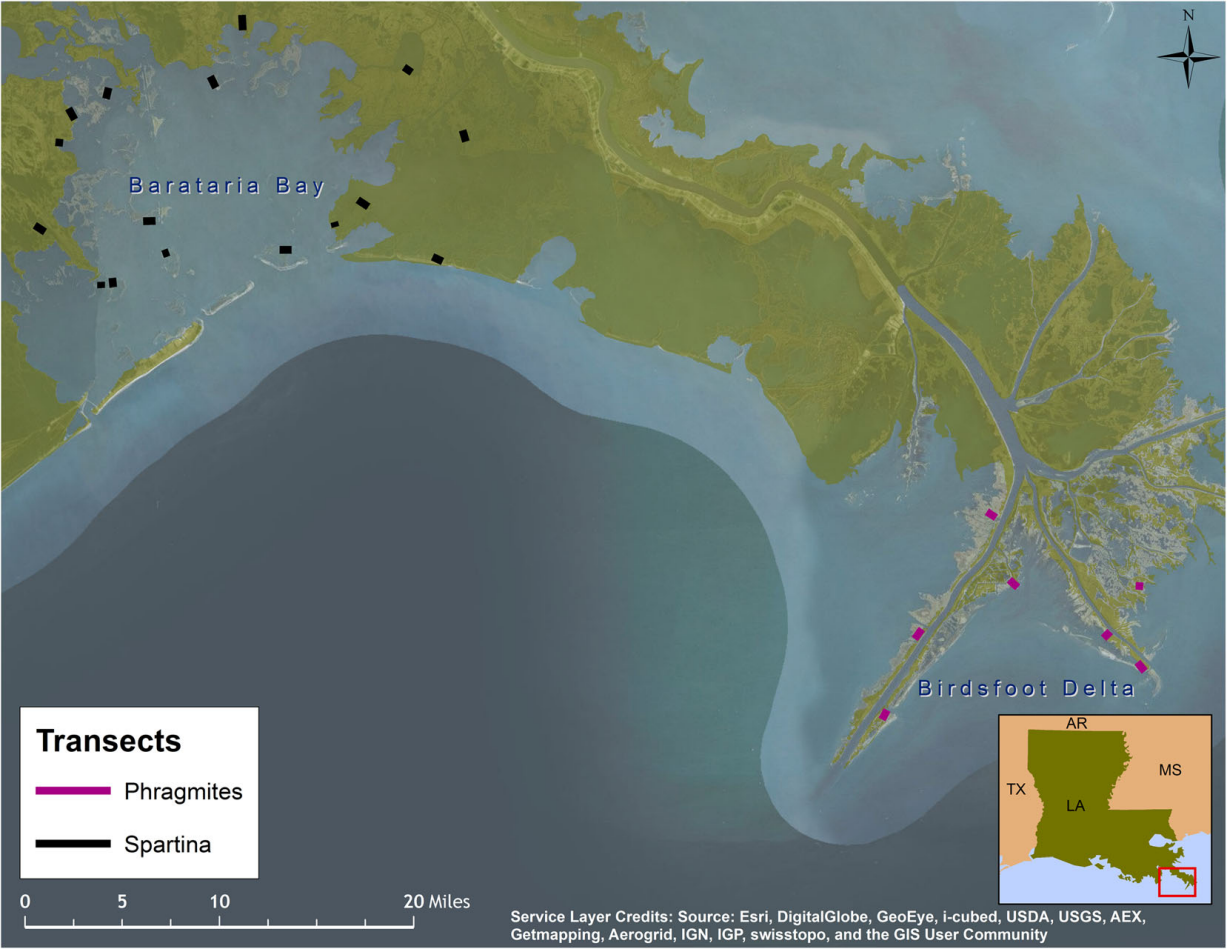
Locations of marsh edge study transects within the study area for the carcass persistence study

Carcass persistence along marsh edges was evaluated in late October and early November 2011 in combination with an evaluation of searcher efficiency/detection probability along marsh edges (Donlan et al. 2013). Each study carcass was uniquely marked with numbered cryptic tags. One hundred seventeen non-oiled, intact bird carcasses of four size categories were placed in random, predetermined locations on 23 transects along marsh edges in two areas in Louisiana: Barataria Bay and the “birdsfoot” of the Mississippi River Delta (Fig. 2). The marsh edges used in the persistence study were located in southeastern Louisiana, since coastal marshes impacted by the spill were generally not located in the eastern half of the zone used for modeling acute mortality. In fact, 95% of the coastal marshes oiled to any degree during the DWH incident occurred in Louisiana (*Deepwater Horizon* Natural Resource Damage Assessment Trustees 2016). Each carcass placement was directed to a specific distance into the marsh vegetation from the vegetation/water interface, up to 3.5 m into the vegetation. However, there was some uncertainty in identifying a distinct “marsh edge” from which to measure distance into marsh, as some marsh edges appeared as a gradient of increasing vegetation density toward the marsh, starting with thinly spaced stems in open water. Two types of marsh edges were investigated: *Spartina*- and *Phragmites*-dominated marshes. Given the presence of standing water at many deployment locations, wooden blocks would not have been effective and thus were not placed under carcasses. Using GPS, each carcass was visited by boat and visually inspected every day after deployment for 11 days, weather permitting, or until the carcass disappeared, whichever occurred sooner. If a carcass was missing from its last known location, field teams searched a larger length of marsh edge to verify the disappearance of the carcass.

### Statistical analyses

#### Sandy beach carcass persistence

We used “nest survival” models (Dinsmore et al. 2002) in program MARK (White and Burnham 1999) to estimate persistence rates from carcass persistence field data. These nest survival models were developed for data where the sample units can be located and their fates determined with certainty even when surveys occurred at different intervals and the exact date on which the fate was achieved may not be known (e.g., sample unit was not surveyed for a day but on the next survey day the fate was discovered). Although our sample units were carcasses (as opposed to “nests”), our objective was to determine the probability that they would persist (“survive”) along the sandy beach transects. Therefore, if a carcass disappeared from the sampling area (e.g., tidally washed out to sea and sunk below the water’s surface or taken by a scavenger), we were able to consider that movement out of the study area a “destroyed nest” in the model. Because investigators knew where all the carcasses were placed within the sampling area and searched for missing carcasses, we assumed that detection of carcasses was 100% (i.e., if a carcass was not seen on a sampling occasion, we concluded it no longer persisted within our sampling frame).

We used model selection (Burnham and Anderson 1998) and parameter estimates to assess whether carcass size, carcass age, and carcass position on the beach influenced daily persistence rates. We conducted our analysis using three carcass size categories (medium, large, and extra-large), which had an acceptable sample size (Table 1). Field investigators used a total of eight small carcasses distributed across the three positions on the beach, which resulted in no or very low numbers of study carcasses in some combinations. Therefore, we excluded the small carcasses from our analysis due to small sample size. Although the large and extra-large size classes were grouped into a single “large” category during the field study, we analyzed them separately. Of the three size categories, we expected the medium carcasses would disappear fastest because smaller carcasses are easier for scavenging animals to move, while the extra-large carcasses would persist the longest. We did not want to assume a linear relationship between size class and persistence, so we analyzed size class as a categorical predictor variable. We expected carcasses along the lower beach to have the lowest persistence rates, because they were closest to the water and presumably more likely to wash away with tidal action. We had no prediction concerning differences in persistence rates between carcasses along the wrack and upper beach and considered position a categorical predictor. Carcasses used for the field study were clean whole carcasses obtained from various sources at various times, and some had been stored frozen until study implementation (IEc 2011). Because we did not know how long ago each individual carcass had been euthanized (i.e., “carcass age”), we used time (i.e., days after deployment) as a surrogate measure of carcass age and predicted that daily persistence rates would increase with days surveyed (i.e., the proportion of carcasses persisting from 1 day to the next would increase through time). We also assumed that the increase in persistence rate would not be linear. Previous research indicated that carcasses rapidly disappear during the first few days and those that are still present after the first few days tend to remain (Byrd et al. 2009; Ford 2006; Ford and Zafonte 2009). We used a natural log transformation of the predictor variable (“days”) to accommodate the non-linear change in persistence rates through time. We compared univariate, additive, and all two-way interaction models with the three predictor variables (i.e., carcass size, position on the beach, and time). We also included intercept-only (i.e., a mean persistence rate) for a total of 18 models.

**Table 1:** Numbers of carcasses included in the analyses of the effect of carcass size and beach position on persistence on sandy beaches; the effect size and marsh type on persistence along marsh edges; and the effect of carcass size, marsh type, and distance into marsh on persistence along marsh edges

	Sandy Beach (*n* = 105)			Marsh type (*n* = 116)		Marsh type (distance)^a^ (*n* = 105)	
Carcass size	Lower beach	Wrack	Upper beach	*Spartina*	*Phragmites*	*Spartina*	*Phragmites*
Small				18	6	14	6
Medium	16	18	18	25	10	20	10
Large	9	14	8	24	7	23	7
Extra Large	9	6	7	17	9	16	9

^a^Sample sizes after limiting marsh edge data to carcasses with reliable distance information

For model selection, we calculated a small sample size–adjusted Akaike’s information criteria(AIC_*c*_) (Burnham and Anderson 1998) to identify the “best model” (i.e., the combination of predictor variables that best fits the carcass persistence study data and has the lowest AIC_*c*_). Although we assumed that any influence of carcass age would be a threshold form, peer reviewers suggested we try linear (i.e., persistence increases in a continuously consistent pattern) and quadratic (i.e., where daily survival would increase to point and then begin to decrease again) functional form of carcass age. We did not a priori assess the linear and quadratic forms, so we post hoc fit a quadratic form on the minimum AIC_*c*_ a priori model to see if the quadratic time model improved model fit.

When a model ranks just below the minimum AIC_*c*_ model and has one additional parameter than the minimum model, inferences regarding the importance of that additional parameter are precluded (Arnold 2010). Therefore, in addition to model selection, we assessed the importance of individual variables within models using 95% confidence intervals (CI) for the parameter estimates. Parameter estimates from the models provide a quantitative measure of the relationship between the response variable (carcass persistence) and each of the predictor variables (carcass size, position, and age/time) while accounting for the possibility that other predictors in the model influence the response variable. If an estimate for a particular parameter was zero, it would indicate that our data failed to detect a correlation between that variable and persistence. If the 95% CI exclude zero for a particular predictor variable, we concluded that we detected a strong relationship between that predictor variable and carcass persistence. Thus, a parameter estimate greater than zero with CI that exclude zero indicates a strong positive relationship between the predictor and response variable, whereas a parameter estimate less than zero with CI that exclude zero indicates a strong negative relationship.

Although we used model-specific parameters to assess correlations between predictor variables and carcass persistence, we used model averaging in program MARK (White and Burnham 1999) to present the fitted daily persistence rates and their unconditional variances (i.e., variances include model selection uncertainty; Burnham and Anderson 1998).

#### Marsh edge carcass persistence

The field study provided data on 116 study carcasses for consideration in the persistence analyses (one carcass was excluded because it was never revisited after deployment) (Table 1). We used the same “nest survival” model, conducted model selection, and assessed parameter inferences and importance within the top models in the same manner as described for the sandy beach analysis. During field implementation, many of the carcasses were checked several hours after deployment, and some carcasses had disappeared by then. We treated these carcasses as missing within 1 day after placement, since data for a shorter time interval were not available for all carcasses. Data were available to determine the fate of most carcasses on each day after carcass deployment. However, there were a few instances when the fate of some carcasses could not be checked for a period of 2 or 3 days. As described above, the “nest survival” models in program MARK accommodate such situations, and it was not necessary to surmise daily fates for these data gaps.

We were interested in evaluating the influences of carcass size, time, and distance into the marsh on daily persistence along marsh edges, as well as comparing persistence in two different marsh-edge habitat types (*Spartina*-dominated vs *Phragmites*-dominated). As in the sandy beach carcass persistence study, we expected the smaller carcasses would disappear fastest, while the extra-large carcasses would persist the longest, but we did not assume a linear relationship and analyzed size as a categorical predictor variable. Although we acknowledged the possibility that different marsh habitat types could contain different types of scavengers and thereby different carcass persistence rates, we had no expectation of which habitat type might have a higher carcass persistence rate. We again used time as a surrogate for carcass age in a non-linear threshold fashion. As with the sandy beach analysis, we considered linear and quadratic forms post hoc based on reviewer comments. We fit single variable, additive and all two-way interaction models for the three variables (i.e., size, habitat type, and time). We also included intercept-only (i.e., a mean persistence rate), for a total of 18 models. We provide model-averaged estimates of daily probability of persistence over all a priori models.

We evaluated whether carcass persistence was influenced by distance from marsh edge into vegetation in a separate analysis because data revealed that deployed carcasses moved around somewhat during the study, and distance data were not consistently collected by investigators after the date of carcass deployment. Therefore, we limited the analysis of the effect of distance on carcass persistence to the first 24 h after deployment (from day 1 to day 2), the period during which the distance data are most reliable, and treated the analysis of distance completely independent of the model selection exercise used for marsh habitat type, time, and carcass size. We removed 11 carcasses from the analysis exploring the influence of distance into the marsh because they either had missing distance data (1 carcass) or their presence 24 h after deployment could not be determined (10 carcasses). Regarding the latter condition, some carcasses were not checked on the second day but were checked on day 3 or later. If one of these carcasses was found present on day 3 or later, we considered the carcass present on day 2; but if the carcass was absent, we could not assume presence or absence for day 2, and these carcasses were excluded for lack of data. A total of 105 carcasses were used in the distance analysis (Table 1). We used logistic regression (glm package in program R) with a logit link to analyze the distance into marsh data. Whether the carcass persisted from day 1 to day 2 was the response variable (persisted = 1, did not persist = 0) and distance into marsh, habitat type, and size class were the predictor variables. We estimated single variable, additive, and all 2-way interaction models for the three variables. We also included intercept-only (i.e., a mean persistence rate), for a total of 18 models. We provide model-averaged estimates of persistence over all a priori models that spanned the distance ranges of the raw data (0–3 m into the marsh).

## Results

### Sandy beach

Considering only the 105 study carcasses deployed on sandy beaches in the medium, large, and extra-large size classes, 32 were considered to have persisted for the entire study. Thirty-one carcasses disappeared within the first 24 h after deployment, the causes of which were tidal action (45%), human intervention (22%), natural scavengers (27%), and undetermined (5%). Forty-eight carcasses disappeared during the first 3 days post-deployment; of these carcasses, 60% disappeared due to tidal action, 27% were due to natural scavenging activity, 8% were due to human intervention, and 4% had unknown causes. For carcasses persisting past the first 3 days, tidal action was less likely to be the cause of later disappearance (16%) than human intervention (48%) or scavenging (28%).

**Fig. 3:**
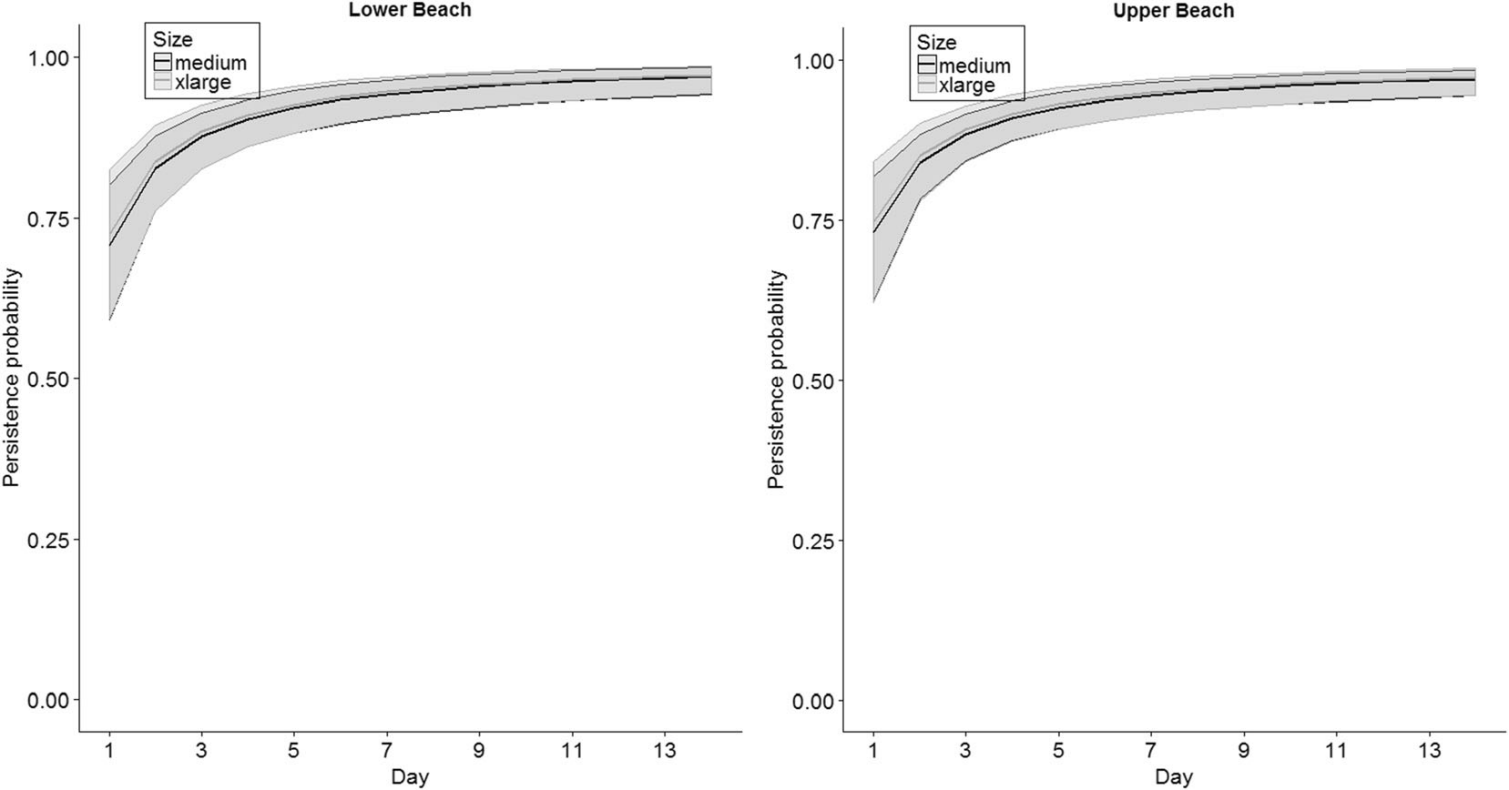
Model-averaged daily persistence probabilities for medium and extra-large carcass size classes and the two extreme beach positions along sandy beaches, northern Gulf of Mexico, June 2011. Shaded areas represent 95% CIs. Note, the large size class and the wrack position were not plotted for simplicity but appeared to have parallel trends intermediate to those shown

The quadratic time effect (AIC_*c*_ = 452.96) and linear time-effect (AIC_*c*_ = 452.18) models did not fit as well as the threshold trend (AIC_*c*_ = 446.58) in the global model, so we present results based on the threshold form of the model. Model selection indicated that time (as a surrogate for carcass age) influenced persistence (Table 2). The influence of time appeared to be strong, because models with the time effect contained 100% of the model weights (Table 2) and 95% confidence intervals for time in the “best model” excluded zero (*β*_Time_ = 0.97, SE = 0.15, 95% CI = 0.67, 1.26). The threshold time effect model indicated that daily persistence rates were relatively low the first day, but rapidly increased and then leveled off (Fig. 3). The second-ranked model included size as a categorical covariate and indicated that persistence probability increased sequentially from medium to large, and to extra-large carcasses. However, the confidence interval for the large and extra-large parameter estimates overlapped zero, which indicated that estimates for those size classes were not statistically different than the medium size (*β*_large_ = 0.01, SE = 0.29, 95% CI = −0.57, 0.58, *β*_xlarge_ = 0.37, SE = 0.35, 95% CI = − 0.32, 1.05, Fig. 3). The highest ranked model that included position on the beach was ranked third and indicated that carcasses on the lower beach persisted for shorter time spans than those on the wrack (*β*_wrack_ = 0.23, SE = 0.31, 95% CI = − 0.37, 0.84) and upper beach (*β*_upper_ = 0.34, SE = 0.32, 95% CI = − 0.29, 0.97). However, the parameter estimates overlapped zero for the position variables, indicating that any effect of position was extremely weak compared with the influence of time.

**Table 2:** Model selection results for study assessing factors that may affect the persistence of bird carcasses along sandy beaches. Size = categorical factor for size of carcass (medium, large, and extra-large); position = categorical factors for lower, wrack, and upper positions on the beach; and ln(T) represents a threshold time effect (using a natural logarithmic transformation of days)

Model^a^	Deviance	Number of parameters	AIC_*c*_	Difference in AIC_*c*_	AIC_*c*_ weight
ln(T)	424.09	2	428.11	0.00	0.58
ln(T) + size	422.80	4	430.86	2.76	0.15
ln(T) + position	422.90	4	430.96	2.85	0.14
ln(T)*position	421.02	6	433.14	5.04	0.05
ln(T) + size + position	421.44	6	433.56	5.45	0.04
ln(T)*size	422.52	6	434.64	6.53	0.02
ln(T)*position + size	419.47	8	435.68	7.57	0.01
ln(T)*size + position	421.16	8	437.37	9.26	0.01
ln(T)*size + ln(T)*position	419.07	10	439.38	11.28	0.00
ln(T) + size*position	420.46	10	440.77	12.67	0.00
ln(T)*position + size*position	418.45	12	442.90	14.80	0.00
ln(T)*size + size*position	420.17	12	444.62	16.52	0.00
ln(T)*size + ln(T)*position + size*position	417.97	14	446.58	18.47	0.00
Intercept only	469.69	1	471.70	43.59	0.00
Position	467.50	3	473.53	45.43	0.00
Size	467.95	3	473.99	45.88	0.00
Size + position	465.57	5	475.66	47.55	0.00
Size*position	464.23	9	482.49	54.39	0.00

^a^+ represents additive effects; * represents interaction effects

In summary, these analyses indicated that daily carcass persistence on sandy beach shorelines increased with time. Carcass size and position on the beach did not strongly influence daily persistence (Fig. 3, numerical values can be found in the Electronic Supplementary Material). The model-averaged estimates of persistence for different sizes and positions on the beach illustrate that any persistence differences among these factors were small given our data. The predominant cause of carcass disappearance within the first few days of the study was tidal action.

### Marsh edge

Of the 116 study carcasses effectively deployed in marsh habitat, at least 50 disappeared within the first 24 h post-deployment. Twelve carcasses persisted for the entire study, and slightly over half of these carcasses were intact or nearly intact birds with little scavenging by the end of the study.

**Fig. 4:**
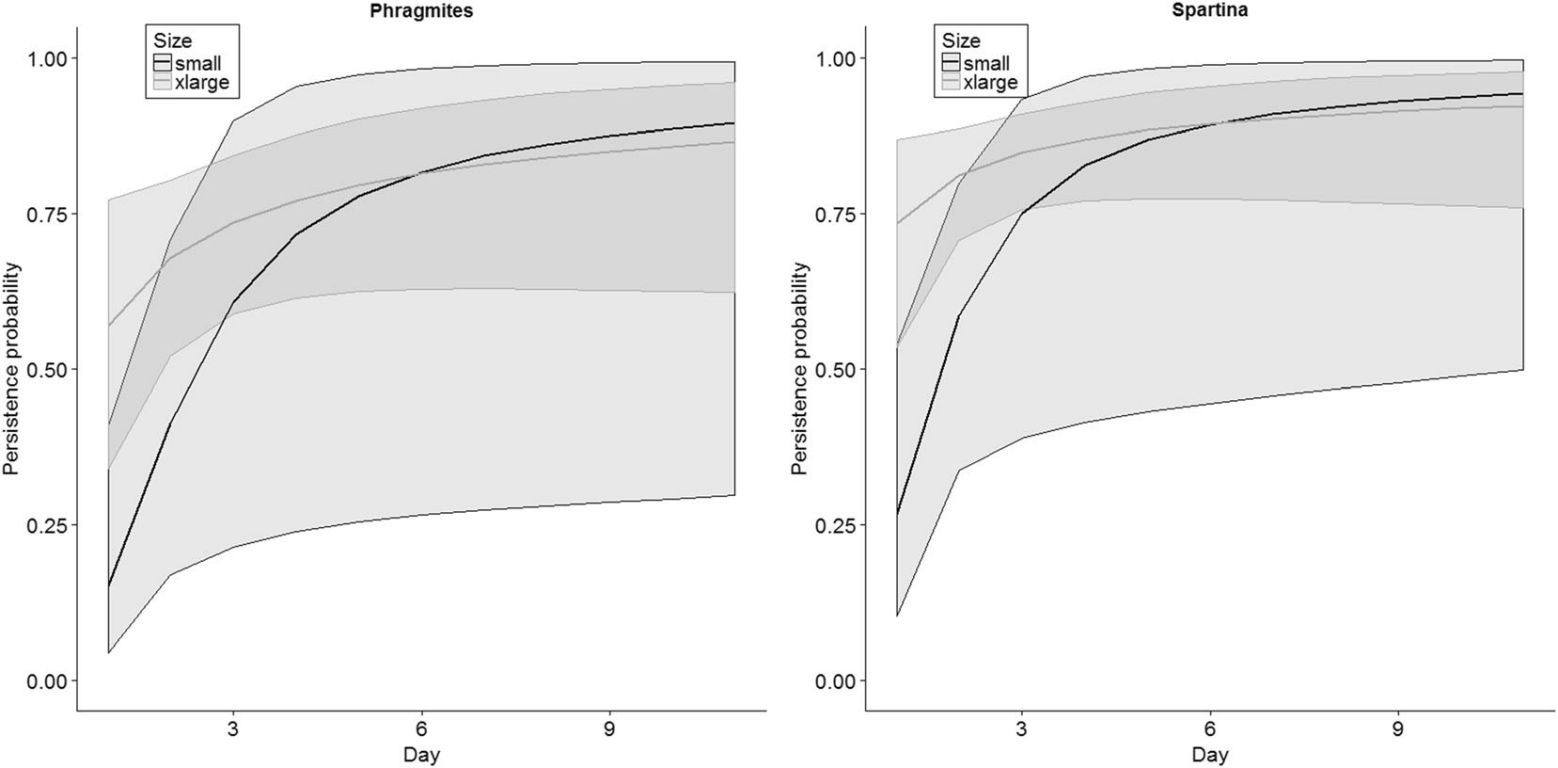
Model-averaged daily persistence probabilities in two marsh habitat types and for the two extreme carcass size classes along the northern Gulf of Mexico, October–November 2011. Shaded areas represent 95% CIs

Models that included an interaction between habitat and size did not converge (Table 3) because all of the small carcasses in *Phragmites* disappeared the first night. Because models with habitat by size interactions did not converge, we considered the model with time by habitat and time by size as the global model for assessing whether quadratic or linear trends fit the data better. The quadratic (AIC_*c*_ = 323.10) and linear (AIC_*c*_ = 324.64) time trend models did not improve the fit compared with the threshold model (AIC_*c*_ = 322.84), so we present our results based on the threshold form of the model. Model selection based on the 13 models that adequately converged indicated that time (as a surrogate for carcass age), habitat type, and carcass size influenced persistence (Table 3). The best model indicated an interaction between time and size with an additive effect of habitat. This model suggested that daily persistence rates increased more rapidly in small carcasses compared with larger ones and that daily persistence rates were slightly higher in *Spartina* habitats compared with *Phragmites* (e.g., see Fig. 4 and Table 4). A second-ranked model also included time, size, and habitat, but indicated that time and size were additive (i.e., persistence rates increased at a similar rate from day 1 to 14 for each size class). A third model was also closely competing with the top model and was very similar except that it included an interaction between habitat and time. However, the interaction between time and habitat was weak (Table 4) and estimates from the top model and this model were very similar. The 95% confidence intervals for the parameter estimates from these top three models indicated strong support for the effects of time, habitat, size class, and an interaction between time and size class (Table 4). Model averaged estimates of daily persistence probabilities followed these relative patterns of larger carcasses having higher daily persistence rates the first few days, smaller carcasses having daily persistence rates increasing more rapidly during the first few days, and overall persistence rates being slightly higher in *Spartina*-dominated marshes (Fig. 4, numerical results can be found in Electronic Supplementary Material).

**Table 3:** Model selection results for study assessing factors that may affect the persistence of bird carcasses along marsh edge shoreline, considering four carcass size categories. Size = categorical factor for size of carcass (small, medium, large, and extra-large); habitat = habitat of marsh transect (*Phragmites*, *Spartina*); and ln(T) represents a threshold time effect (using a natural logarithmic transformation of days)

Model^a^	Deviance	Number of parameters	AIC_*c*_	Difference in AIC_*c*_	AIC_*c*_ weight
ln(T)*size + habitat	302.54	9	321.07	0.00	0.41
ln(T) + habitat + size	309.87	6	322.12	1.05	0.24
ln(T)*habitat + ln(T)*size	302.19	10	322.84	1.77	0.17
ln(T)*habitat + size	309.86	7	324.19	3.12	0.09
ln(T)*size	308.56	8	324.98	3.92	0.06
ln(T) + size	316.79	5	326.96	5.90	0.02
ln(T) + habitat	322.34	3	328.41	7.35	0.01
ln(T)*habitat	322.32	4	330.44	9.37	0.00
ln(T)	327.84	2	331.87	10.81	0.00
Habitat + size	342.31	5	352.48	31.42	0.00
Size	352.08	4	360.19	39.12	0.00
Habitat	364.54	2	368.58	47.51	0.00
Intercept only	372.32	1	374.34	53.27	0.00
ln(T) + habitat*size^b^	–	–	–	–	–
ln(T)*size + habitat*size^b^	–	–	–	–	–
ln(T)*habitat + habitat*size^b^	–	–	–	–	–
ln(T)*habitat + ln(T)*size + size*habitat^b^	–	–	–	–	–
Habitat*size^b^	–	–	–	–	–

^a^+ represents additive effects; * represents interaction effects

^b^Model did not converge

**Table 4:** Regression parameter estimates for the top ranked models (< 2 AIC*c* units) used to assess the influence of time, habitat, and carcass size on persistence rates of carcasses along marsh edges. N/A represents parameters that are not included in that particular model

	First-ranked model: ln(T)*size + habitat		Second-ranked model: ln(T) + habitat + size		Third-ranked model: ln(T)*habitat + ln(T)*size	
Parameter	Estimate	SE	Estimate	SE	Estimate	SE
Intercept	− 2.06^a^	0.59	− 1.41^a^	0.45	− 2.21^a^	0.65
ln(T)	2.58^a^	0.94	0.99^a^	0.19	2.80^a^	1.01
Habitat	0.76^a^	0.31	0.80^a^	0.30	0.93^a^	0.43
Medium	1.51^a^	0.63	0.85^a^	0.43	1.54^a^	0.63
Large	1.97^a^	0.63	1.34^a^	0.43	1.98^a^	0.64
Xlarge	2.51^a^	0.66	1.37^a^	0.44	2.55^a^	0.67
ln(T)*habitat	N/A	N/A	N/A	N/A	− 0.24	0.41
ln(T)*medium	− 1.56	1.01	N/A	N/A	− 1.62	1.01
ln(T)*large	−1.51	1.00	N/A	N/A	− 1.53	1.00
ln(T)*xlarge	−2.13^a^	1.00	N/A	N/A	− 2.19^a^	1.00

^a^Confidence interval excludes zero indicating a correlation between variable and persistence

**Fig. 5:**
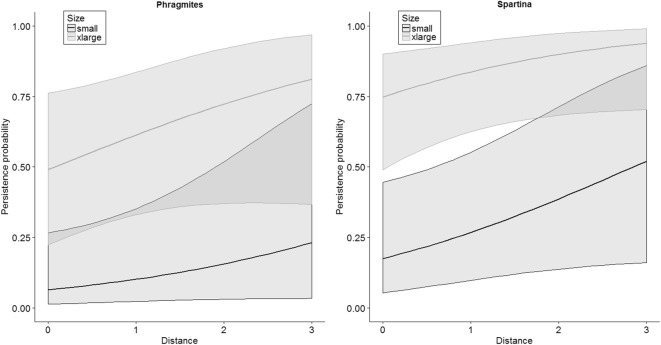
Model-averaged daily persistence probabilities as a function of distance into marsh within two marsh habitat types and for the two extreme carcass size classes along the northern Gulf of Mexico, October–November 2011. Shaded areas represent 95% CIs. Note, the intermediate (i.e., medium and large) size classes were not plotted for simplicity and had parallel trends intermediate to the small and extra-large size classes

Model selection on the subset of data with distance into marsh indicated that distance into the marsh, carcass size, and habitat all correlated with carcass persistence over the first 24 h (Table 5). This model suggested that carcass persistence during the first day increased as carcasses were placed further into the marsh (from approximately the edge to 3 m into the marsh), carcass persistence increased with size class and was slightly higher in *Spartina*-dominated marshes (Fig. 5).

**Table 5:** Model selection results for exploring the influence of distance into marsh, habitat, and carcass size on carcass persistence during the first 24 h after being placed in the marsh. Size = categorical factor for size of carcass (small, medium, large, and extra-large); habitat = habitat of transect (*Phragmites*, *Spartina*); and distance = distance of the carcass into the marsh from the marsh edge (range = 0–2.9 m, where 0 = at the marsh open water interface)

Model^a^	Deviance	Number of parameters	AIC_*c*_	Difference in AIC_*c*_	AIC_*c*_ weight
Distance + size + habitat	117.87	6	130.73	0.00	0.55
Distance*habitat + size	117.68	7	132.83	2.10	0.19
Size + habitat	124.30	5	134.91	4.18	0.07
Distance + size	124.52	5	135.12	4.40	0.06
Distance*size + habitat	117.44	9	137.34	6.61	0.02
Distance + habitat	133.10	3	139.33	8.61	0.01
Distance*size + distance*habitat	117.10	10	139.44	8.72	0.01
Size	131.63	4	140.03	9.31	0.01
Distance * habitat	133.09	4	141.49	10.76	0.00
Distance*size	124.35	8	141.85	11.12	0.00
Distance	138.40	2	142.52	11.79	0.00
Habitat	139.28	2	143.40	12.67	0.00
Mean	145.32	1	147.36	16.64	0.00
Distance + habitat*size^b^	–	–	–	–	–
Distance *size + habitat*size^b^	–	–	–	–	–
Distance *habitat + habitat*size^b^	–	–	–	–	–
Distance *habitat + distance *size + size*habitat^b^	–	–	–	–	–
Habitat*size^b^	–	–	–	–	–

^a^+ represents additive effects; * represents interaction effects;

^b^Model did not converge

In summary, these data indicated that daily carcass persistence on marsh edges increased with time and carcass size, particularly during the first few days of being deposited in the marshes. In addition, within each size class, persistence appeared slightly higher in *Spartina*-dominated habitats than in *Phragmites* habitats. We found some evidence carcasses further into the marsh from the edge had higher persistence rates during the first day.

## Discussion

The number of days after carcass deployment (i.e., surrogate for carcass age) was an influential factor on carcass persistence in both sandy beach and marsh edge environments. Carcass size and position on the beach did not correlate with persistence along sandy beaches. For marsh edges, persistence rates increased more rapidly over time for small carcasses and appeared to be longer in *Spartina*-dominated habitat than in *Phragmites*-dominated habitat. However, the wide confidence intervals for the model-averaged persistence estimates, particularly for *Phragmites*-dominated marsh, indicated a high degree of uncertainty in the persistence estimates for each size and marsh type combination.

For sandy beaches in the DWH study, the effect of carcass age (time) on daily persistence was consistent with previous studies in which carcasses rapidly disappeared during the first few days and the daily persistence probability quickly increased to an asymptote (Byrd et al. 2009; Ford 2006; Ford and Zafonte 2009; Van Pelt and Piatt 1995). It has been surmised that change in persistence rate may be partly related to scavenger satiation and/or a reduction in attraction of scavengers to decomposed or mummified carcasses (Fowler and Flint 1997; Van Pelt and Piatt 1995). However, the initial high rate of carcass disappearance in the DWH study on sandy beaches was presumed primarily due to tidal action, evidenced by the simultaneous disappearance of the study carcass and the wooden block and the lack of evidence suggesting other causes. A full moon and spring tide occurred on June 15, 2011 (day 3 post-deployment for over three-quarters of the study carcasses), and correspondingly, the high tides were generally increasing during the first few days of the study and were decreasing after the full moon. Our results suggest that rising tides carry freshly deposited avian carcasses out to sea rather than push them further inland. The higher tidal reach of the spring tide apparently affected several of the study carcasses regardless of their positions on the beach, explaining the very weak relationship between position and persistence identified by our analysis. The influence of tidal action in the northern Gulf of Mexico is in contrast to an Alaska study which experienced little influence from tidal action on persistence (Byrd et al. 2009). However, approximately two-thirds of the study carcasses in the Alaska study were deployed during a time of decreasing high tides. Persistence studies longer than 14 days could further evaluate the relationship of tidal action to persistence. In addition, to more precisely investigate tidal action, the heights of high tides should be measured daily at the location of each study carcass. Tidal information specific to our study sites was not available, so we considered information from nearby tidal observation stations, but this option might not be available or suitable in other study areas.

Ford (2006) concluded that tidal influences on carcass persistence are highly site-specific. We add that tidal influences are also dependent on the stage of the tidal cycle, as tidal height gradually increases over the seven-day periods between first quarter moon and full moon and between last quarter moon and new moon. Despite the recognition by many that tidal action may play a role in carcass persistence rates, most studies do not quantitatively report on its contribution. The magnitude of the contribution of tidal action to site-specific carcass persistence has historically not been explicitly considered when incorporating a persistence probability in mathematical modeling of total carcass deposition. However, this knowledge could be an important factor to consider when designing site-specific carcass persistence studies on sandy beaches to ensure that study conditions, such as tidal cycle, reasonably duplicate conditions during the avian mortality event being modeled. Matching tidal conditions could be particularly important for mortality events that span a time period shorter than the local tidal cycle. Our sandy beach study spanned 14 days and included rising high tides, then falling high tides, and rising high tides again. Had we started our sandy beach study during a period of declining high tides, we expect that the proportion of carcass disappearances caused by tidal action would have been reduced. However, it is possible that the persistence rates could still be similar to our study results, as carcasses not removed by tides would be available for removal by scavengers. The DWH incident spanned many tidal cycles, and therefore we believe our sandy beach persistence values are appropriate for use in the Trustees’ modeling of carcass deposition.

Ford et al. (2006) found that, due to increased buoyancy, partially scavenged/decomposed carcasses were more likely to be washed out to sea than intact carcasses. It is possible that scavenging activity also played a role in the disappearance of the sandy beach study carcasses during the first few days of the DWH study, increasing the buoyancy of the study carcasses and increasing their vulnerability to tidal action. At 24 h after deployment, approximately half of the study carcasses that remained on the beach were beginning to experience some scavenging. By 3 days after deployment, only eight out of the 113 carcasses deployed (small size class included) remained intact on the beach.

Human intervention and natural scavenging activity were the predominant causes of carcass disappearance for carcasses that remained on sandy beaches past 3 days post-deployment. During days 4 through 11 post-deployment, the high tides were decreasing in height, so any carcasses remaining by day 3 were less likely to be affected by tides. The human intervention is reflective of the many recreational beaches comprising the northern Gulf of Mexico.

Our analysis of the sandy beach study revealed no correlation between carcass size and persistence, in contrast to other studies that evaluated the effect of carcass size on persistence and found stronger evidence that larger carcasses persist longer (Ford et al. 2001, 2006, and 2009; Ford and Zafonte 2009). Our finding may be associated with the rising tides that occurred during the first few days of the study carrying away carcasses of all size classes nearly equally, much like the tides may have removed carcasses regardless of their position on the beach.

Persistence on the sandy beaches of this study is likely influenced by tidal action, scavenging animals, and human interference. We considered whether the spatial scale of the sandy beach study, spanning from western Louisiana to western Florida, and geographic variations in tides, scavengers, and humans may have confounded our persistence values. Outside of large storm events, the tidal height does not notably vary among sites within the northern Gulf of Mexico (NOAA 2011). The presence and feeding activities of scavengers on the sandy beach study transects may be affected by characteristics of the transects themselves, such as the degree of rural and urban development inland of the shoreline transect, the proximity of the transect to scavenger congregation areas, and the availability of food sources that are more preferred over carrion. These factors are present throughout the study area, but we have no quantitative information that would allow us to evaluate those factors. The proportion of sandy beaches that are recreational beaches is generally less in Louisiana than in the rest of the study area; thus, the probability of human intervention affecting persistence may be lower in Louisiana. If one assumes that all else is equal, this suggests that persistence in Louisiana could be relatively higher than in the rest of the study area. A comparison of raw data reveals that 42% of the carcasses deployed in Louisiana (*n* = 24, excluding small carcasses) persisted through the entire study period, compared with 27% in the rest of the study area (*n* = 81, excluding small carcasses). However, of those carcasses that disappeared, the average time to disappearance was 3.2 days (SE = 0.8) for Louisiana and 3.7 days (SE = 0.5) for the rest of the study area. Despite the potential for geographical variability in persistence, the choice of study locations in sandy beaches was appropriate for the area impacted by the spill and for the intended use of the persistence results (i.e., modeling acute mortality in one large-scale model).

Persistence rates measured by other investigators of avian carcasses on sandy beaches have shown to vary greatly. The proportions of carcasses of similar size category to our “medium” size class that remained on sandy beaches after 2 days post-deployment/stranding ranged from 0.1 to 0.9 (Byrd et al. 2009; Ford et al. 1991, 2001, 2002, 2006, 2009; Fowler and Flint 1997; Page and Carter 1986; Van Pelt and Piatt 1995). These studies were conducted along coastlines in Alaska, Washington, Oregon, and California. The DWH study observed that approximately 0.55 of the medium-sized carcasses remained after 2 days. To our knowledge, this was the first, and so far only, study to provide avian carcass persistence information for the northern Gulf of Mexico. The high variability among studies, as well as our analyses of factors influential to persistence in just one geographic area, highlight the importance of obtaining site-specific persistence information for each mortality incident when the total number of mortalities will be estimated from carcasses collected from shorelines.

Avian carcass persistence in marshes appears to be less studied than for sandy beaches and are primarily focused on non-coastal freshwater wetlands in association with evaluating bird collisions with human-made structures or pesticide poisoning events. The observed effect of carcass age on persistence found in the DWH study in estuarine marshes was contrary to studies in freshwater emergent marshes, which generally did not find a significant increase in daily persistence probability after the first few days (Goldberg et al. 2004; Linz et al. 1991). In the DWH marsh study, the cause of carcass disappearance (e.g., tidal action or scavenging) could not be studied directly. However, the intact or near-intact conditions of slightly over half of the carcasses that persisted to 11 days post-deployment suggest that natural composition and scavenging in situ were not likely major contributors to carcass disappearance. Carcass disappearance may have occurred mostly through whole-carcass removal events, involving either scavengers large enough to remove an entire carcass at once or tidal action washing carcasses out to sea. Unlike for sandy beaches, inferring the role of the tidal cycle on the marsh study results is complicated by the fact that not all marsh transects began the study at the same stage of the tidal cycle.

Our marsh edge analyses revealed a positive correlation between carcass size and persistence. Ford and Zafonte (2009) found little support for an effect of size on persistence at one of the California coastal marsh sites tested but found significant size effect at the second marsh studied (equal sample sizes). The freshwater marsh studies did not evaluate an effect of size, as their study carcasses did not vary in size (Goldberg et al. 2004, Linz et al. 1991).

Our analyses found evidence for small differences in persistence between *Spartina-* and *Phragmites-*dominated habitats, with slightly higher persistence in *Spartina* marshes within carcass size classes. We thought a priori that a difference in persistence could be due to potential differences in scavengers between habitat types. After further consideration, we have not found any information that would support major differences in scavenger types or numbers between the two marsh habitat types in our study. We considered the different geographical locations of the marsh edge study transects; the *Spartina* transects were located in Barataria Bay, while the *Phragmites* transects were located on the “birdsfoot” of the Mississippi River Delta approximately 60 km southeast of Barataria Bay. Perhaps Barataria Bay and the birdsfoot experienced different environmental temperatures, as the marsh edge field study occurred during the onset of fall. Carcass persistence is suspected to vary among seasons, with changes in scavenger presence or behavior (Byrd et al. 2009; Ford and Zafonte 2009; Ford 2006; Fowler and Flint 1997). The marsh edge study occurred during the time of year that American alligators would usually be entering winter dormancy and associated fasting. Available water temperature data for the regions are sparse but indicate that temperatures during the study were likely above the 16 °C threshold for fasting at all of the marsh transects during the periods that they were surveyed (Lance 2003; NOAA 2011). Thus, seasonal influences do not appear to be responsible for the difference in persistence between *Spartina*- and *Phragmites*-dominated marshes in our study.

The difference in persistence may be associated with a greater density of vegetation at the ground/water level in *Spartina* marshes than in *Phragmites*. Specifically, *Phragmites*-dominated marshes may have more open space between stems than *Spartina*-dominated ones, allowing scavengers to more easily move through the vegetation and locate carcasses. The field investigators did not collect vegetation density data during the marsh edge persistence study, but inference can be made from the marsh edge searcher efficiency study results (Zimmerman and Varela 2020), in which it was easier to visually detect carcasses in *Phragmites* than in *Spartina*, presumably because of the lesser density of vegetation in *Phragmites*. In both marsh types, vegetation density generally increased with distance from the marsh edge into the marsh. Our analyses found that persistence within the first 24 h increased with increasing distance into the marsh (up to 3 m). Thus, vegetation density may play a role in marsh carcass persistence.

In general, within a carcass size class, carcass persistence on sandy beaches was greater than on marsh edges. This is contrary to the findings of the California study which discovered significantly higher persistence for birds < 521 g at two marsh study sites (46.7% remaining on beach after 4 days) compared with four sandy beach sites (2.6% remaining on beach after for days) (Ford et al. 2002; Ford and Zafonte 2009). The difference was attributed to site-specific variations in scavengers large enough to physically remove entire study carcasses. In the northern Gulf of Mexico, the lower persistence along the marshes of Louisiana compared with beaches throughout the region may be attributable to the presence of greater numbers of scavengers, which include, in addition to avian and mammalian scavengers also common to sandy beaches, American alligators, alligator snapping turtles (*Macrochelys temminckii*), and common snapping turtles (*Chelydra serpentina*), which are frequently found in estuarine marshes but rarely found on sandy coastal beaches. Louisiana’s coastal marshes are home to the largest population of alligators in the southeastern USA. Considering that the DWH beach and marsh studies took place at different times of the year, the difference between sandy beach and marsh edge persistence could also be due to seasonal variations. For example, herring gulls (*Larus argentatus*), one of the potential scavengers at both beaches and marshes, are somewhat less abundant in the northern Gulf of Mexico during June compared with the autumn months (eBird 2012), and this could contribute to a greater carcass persistence in June at both habitat types. On the other hand, scavenger bird species are busy rearing chicks during the summer, and an increased need for food for chick provisioning could contribute to a decrease in carcass persistence. Since the contribution of each scavenging species to the carcass persistence observed in the DWH studies is not known, it is not possible to determine what effect, if any, seasonal differences in scavenging had on our results. There may be seasonal increases in the presence of human beach-goers on sandy beaches during warmer months potentially decreasing carcass persistence, but such seasonal effect on persistence would not occur at marsh edges, as marsh edges seldom experience the magnitude of recreational use that sandy beaches receive. Differences in maximum tidal heights among the seasons appear negligible for the northern Gulf of Mexico (NOAA 2011). During the DWH incident, the majority of bird carcasses were collected May through September 2010, and this was the time period considered by the Trustees for modeling total acute avian mortality (Amend et al. 2020). We believe the sandy beach carcass persistence study provides unbiased results with respect to seasonal variability for purposes of the model. We believe the marsh edge study also provided relevant persistence values although the study was performed outside the model period.

We do not believe that our results were affected by a potential preference of scavengers for non-oiled food items over oiled items. We know of no scientific studies evaluating whether oiled bird carcasses in the field are less palatable to, and thus avoided by, scavenging animals. Scant information gleaned from published toxicological studies that fed animals oil-contaminated food ad libitum suggests that avoidance of oil-contaminated food may occur and likely varies with species and gender of the scavenger (Beckett et al. 2002; Horak et al. 2017; Pattee and Franson 1982; Stubblefield et al. 1995a, 1995b; White et al. 1991). However, information could not be found specific to most of the species likely to be scavengers along the shorelines of the northern Gulf of Mexico: American alligator, alligator snapping turtle, ravens, crows, vultures, coyote, fox, raccoon, sharks, shoreline invertebrates, and insects, among others. In fact, partially scavenged oiled bird carcasses are frequent occurrences at oil spills nationwide (U.S. Fish and Wildlife Service oil spill response specialists P. McGowan, P. Ramirez, D. Sparks, and V. Varela, personal communications, September, 27, 2017). For example, after the 2004 M/V *Selendang Ayu* oil spill in the Aleutian Islands of Alaska, the vast majority of retrieved oiled bird carcasses were partial carcasses (unpublished data). Fowler and Flint (1997) used the oiled bird carcasses that beached during the M/V *Citrus* oil spill as the subjects of their carcass persistence study, and they found that arctic foxes (*Volpes lagopus*) would indeed scavenge the carcasses. Of the carcasses collected during the DWH incident, nearly 40% of all visibly-oiled bird carcasses were found scavenged (unpublished data). Therefore, although some scavengers could avoid oiled carrion, insufficient information is available to provide insights on the impact of such behavior, if any, on our study results. Given the frequency with which scavenged oiled carcasses are typically encountered during oil spills, we do not believe that the DWH studies were adversely impacted by scavenger aversion to oiled prey.

Overdispersion is common with field data (Burnham and Anderson 1998), but estimating overdispersion for our type of data is difficult (Dinsmore et al. 2002). Overdispersion in our data could arise from unmodeled sources of variation in daily persistence rates (i.e., factors other than habitat, carcass size, or alternative trends in daily rates) or a lack of independence among carcasses. Thus, we acknowledge that we could have underestimated variances in our parameter estimates, and model selection could have selected more complex models than our data supported (Richards 2008). We do not believe this would have influenced our results for the sandy beaches since a fairly simple model (no size or position on the beach covariates) was ranked the highest without adjusting for overdispersion. However, accounting for overdispersion in the marsh edge analysis could have resulted in less support for size and habitat given the observed uncertainty associated with confidence intervals for the fitted values.

## Conclusions

To our knowledge, these field studies and our analyses provide the first estimates of avian carcass persistence on sandy beaches and along estuarine marsh edges in the northern Gulf of Mexico. Despite the intuition that larger carcasses and carcasses located above the intertidal zone should persist longer, we found that carcass size and position on the beach had no or little correlation with persistence on sandy beaches, and this may be due to the increasing high tides experienced during the first few days of the study. Carcass size had a stronger influence in marsh edge habitat. Within marsh habitat, there were slightly higher persistence rates in *Spartina*-dominated marshes compared with *Phragmites*-dominated marshes. The most significant influence on carcass persistence rates, regardless of shoreline type, was the amount of time a carcass spent stranded on a shoreline. At both sandy beaches and marsh edges, daily persistence rates increased over time but increased most rapidly during the first few days after carcass deployment. The sandy beach study, and perhaps the marsh edge study, was affected by tidal activity that removed carcasses from the shoreline, and this highlights the potential importance of controlling for environmental conditions during study design.

## Electronic supplementary material

ESM 1 (PDF 153 kb)
