# Supplementary material for: Persistence of avian carcasses on sandy beaches and marsh edges in the northern Gulf of Mexico
Source: Environ Monit Assess. 2020 Mar 17;191(Suppl 4):815. doi: 10.1007/s10661-019-7920-3 (PMC7078173; doi:10.1007/s10661-019-7920-3)
Supplement: Supplementary file 1 — (PDF 153 kb) [file 10661_2019_7920_MOESM1_ESM.pdf]

Electronic Supplementary Material
associated with
Manuscript proposed for *Environmental Monitoring and Assessment*

Persistence of Avian Carcasses on Sandy Beaches and Marsh Edges in the Northern Gulf of Mexico

Veronica W. Varela (corresponding author)
Natural Resource Damage Assessment and Restoration Program
U.S. Fish and Wildlife Service
1011 E Tudor Rd, MS 361
Anchorage, Alaska 99503
Veronica_Varela@fws.gov
(907) 786-3866 office
(907) 786-3848 fax

Guthrie S. Zimmerman
Division of Migratory Bird Management
U.S. Fish and Wildlife Service
Sacramento, California 95819

Introduction

SANDY BEACH CARCASS PERSISTENCE
Model-averaged estimates of daily persistence probability for bird carcasses on sandy beaches in the northern Gulf of Mexico, for each combination of time, position, and size.

Carcass Size	Position on Beach	Day	Estimate	Standard Error	Lower Confidence Interval	Upper Confidence Interval
medium	low	1	0.7076719	0.0542451	0.591498	0.8018736
medium	low	2	0.8266612	0.029693	0.7606045	0.8774282
medium	low	3	0.8764254	0.0219877	0.8265098	0.9134829
medium	low	4	0.9038265	0.0186301	0.8606146	0.9346591
medium	low	5	0.9211963	0.0166886	0.8816477	0.9483042
medium	low	6	0.9332013	0.0153366	0.8961092	0.9576759
medium	low	7	0.9420001	0.0142899	0.9067793	0.9644356
medium	low	8	0.9487289	0.0134303	0.9150436	0.9695031
medium	low	9	0.9540433	0.0126995	0.9216733	0.9734216
medium	low	10	0.9583481	0.0120642	0.927135	0.9765289
medium	low	11	0.9619068	0.0115035	0.931729	0.9790451
medium	low	12	0.9648987	0.0110031	0.9356579	0.9811189
medium	low	13	0.9674494	0.0105526	0.9390648	0.9828534
medium	low	14	0.9696503	0.0101442	0.9420526	0.9843232
medium	wrack	1	0.7222695	0.0462009	0.623475	0.8033191
medium	wrack	2	0.8357139	0.0241757	0.7827137	0.8778045
medium	wrack	3	0.8827927	0.0178121	0.8431344	0.9134539
medium	wrack	4	0.9086776	0.0152497	0.8740667	0.9344891
medium	wrack	5	0.9250872	0.0138377	0.8930423	0.9480895
medium	wrack	6	0.9364348	0.012865	0.9060559	0.9574514
medium	wrack	7	0.9447575	0.0121046	0.9156465	0.9642147
medium	wrack	8	0.9511268	0.0114694	0.9230706	0.9692913
medium	wrack	9	0.9561607	0.0109195	0.9290253	0.9732206
medium	wrack	10	0.960241	0.0104337	0.9339311	0.9763393
medium	wrack	11	0.9636164	0.0099985	0.938058	0.9788665
medium	wrack	12	0.9664557	0.0096052	0.9415883	0.9809506
medium	wrack	13	0.9688778	0.0092472	0.9446503	0.9826948
medium	wrack	14	0.9709687	0.0089195	0.9473364	0.9841734
medium	upper	1	0.7311168	0.0501986	0.6224133	0.817692
medium	upper	2	0.8399286	0.0258467	0.7826034	0.8843711
medium	upper	3	0.8851545	0.0185254	0.8435645	0.9167782
medium	upper	4	0.9100888	0.0156214	0.8744116	0.9363684
medium	upper	5	0.925939	0.0141381	0.893007	0.9493101
medium	upper	6	0.9369277	0.0131995	0.905555	0.9583582
medium	upper	7	0.9450059	0.0125098	0.9146801	0.9649657
medium	upper	8	0.9512016	0.0119546	0.9216684	0.9699628
medium	upper	9	0.9561081	0.0114832	0.9272246	0.9738514

Model-averaged estimates (cont.)

Carcass Size	Position on Beach	Day	Estimate	Standard Error	Lower Confidence Interval	Upper Confidence Interval
medium	upper	10	0.9600926	0.0110699	0.9317689	0.9769496
medium	upper	11	0.9633944	0.0107005	0.9355683	0.979467
medium	upper	12	0.9661765	0.0103661	0.9388009	0.9815472
medium	upper	13	0.9685534	0.0100604	0.9415916	0.9832903
medium	upper	14	0.9706084	0.0097793	0.9440293	0.9847694
large	low	1	0.7089732	0.0566192	0.5872377	0.806628
large	low	2	0.8270116	0.0316268	0.7560787	0.8805753
large	low	3	0.876439	0.0234941	0.8225782	0.9156262
large	low	4	0.9036914	0.0198318	0.8572109	0.9361683
large	low	5	0.9209875	0.0176916	0.8786095	0.9494228
large	low	6	0.9329539	0.0162086	0.8933179	0.9585472
large	low	7	0.9417323	0.015072	0.904159	0.9651433
large	low	8	0.9484508	0.0141479	0.9125461	0.9700977
large	low	9	0.9537609	0.0133688	0.9192671	0.9739348
large	low	10	0.9580649	0.0126962	0.9247989	0.9769816
large	low	11	0.9616251	0.0121057	0.9294483	0.9794513
large	low	12	0.9646198	0.0115809	0.9334223	0.9814884
large	low	13	0.9671742	0.0111099	0.9368663	0.9831935
large	low	14	0.9693793	0.0106839	0.9398858	0.9846391
large	wrack	1	0.7235154	0.0481969	0.6200437	0.8075548
large	wrack	2	0.8360423	0.0257036	0.7792916	0.8804395
large	wrack	3	0.8828025	0.0189615	0.8402423	0.9151692
large	wrack	4	0.9085474	0.01615	0.8715866	0.9356552
large	wrack	5	0.9248877	0.0145861	0.8908219	0.9489335
large	wrack	6	0.9361989	0.0135191	0.9039964	0.9581003
large	wrack	7	0.9445022	0.0126972	0.9136896	0.9647393
large	wrack	8	0.9508617	0.0120198	0.921182	0.9697325
large	wrack	9	0.9558915	0.0114394	0.9271839	0.9736034
large	wrack	10	0.9599711	0.0109305	0.9321236	0.9766795
large	wrack	11	0.9633477	0.0104773	0.9362757	0.9791745
large	wrack	12	0.9661897	0.0100693	0.9398255	0.9812336
large	wrack	13	0.9686152	0.009699	0.9429029	0.9829579
large	wrack	14	0.9707101	0.0093608	0.9456016	0.9844203
large	upper	1	0.7323882	0.0523423	0.6185361	0.8220367
large	upper	2	0.8402885	0.0275931	0.7785761	0.8872911
large	upper	3	0.8851923	0.0199177	0.8400243	0.9188397
large	upper	4	0.9099817	0.01675	0.8713145	0.9378589
large	upper	5	0.9257579	0.0150896	0.8902177	0.9504332
large	upper	6	0.9367062	0.0140335	0.902968	0.959243
large	upper	7	0.9447617	0.0132639	0.9122259	0.9656909

Model-averaged estimates (cont.)

Carcass Size	Position on Beach	Day	Estimate	Standard Error	Lower Confidence Interval	Upper Confidence Interval
large	upper	8	0.9509448	0.0126522	0.919303	0.9705767
large	upper	9	0.9558447	0.0121393	0.9249194	0.9743849
large	upper	10	0.9598263	0.0116945	0.9295048	0.9774227
large	upper	11	0.9631277	0.0113004	0.9333322	0.9798937
large	upper	12	0.9659109	0.0109461	0.9365839	0.9819371
large	upper	13	0.9682899	0.010624	0.9393871	0.9836505
large	upper	14	0.9703476	0.0103291	0.9418326	0.985105
xlarge	low	1	0.7246684	0.0599593	0.593568	0.8258852
xlarge	low	2	0.8380033	0.0336841	0.7608089	0.8937635
xlarge	low	3	0.8848422	0.0247805	0.8267043	0.9252396
xlarge	low	4	0.9105044	0.0205672	0.8611772	0.9434553
xlarge	low	5	0.9267279	0.0180526	0.8825082	0.9551507
xlarge	low	6	0.9379221	0.0163156	0.8971585	0.963191
xlarge	low	7	0.9461173	0.0150037	0.9079376	0.9690038
xlarge	low	8	0.9523796	0.0139559	0.916259	0.9733727
xlarge	low	9	0.9573226	0.0130878	0.9229133	0.9767593
xlarge	low	10	0.9613247	0.01235	0.9283793	0.9794507
xlarge	low	11	0.964632	0.0117111	0.9329647	0.9816343
xlarge	low	12	0.9674118	0.01115	0.9368773	0.9834369
xlarge	low	13	0.9697812	0.0106517	0.9402629	0.984947
xlarge	low	14	0.9718253	0.0102051	0.9432268	0.9862281
xlarge	wrack	1	0.7387433	0.0539027	0.6205883	0.8301723
xlarge	wrack	2	0.8465431	0.0298086	0.7786819	0.8963658
xlarge	wrack	3	0.8908012	0.0218246	0.840131	0.9268106
xlarge	wrack	4	0.9150265	0.0180973	0.8721849	0.9444233
xlarge	wrack	5	0.9303468	0.0159044	0.891966	0.9557684
xlarge	wrack	6	0.9409251	0.0144064	0.9055157	0.9635978
xlarge	wrack	7	0.9486756	0.0132824	0.9154618	0.9692781
xlarge	wrack	8	0.9546027	0.0123875	0.9231252	0.9735605
xlarge	wrack	9	0.9592847	0.0116466	0.9292438	0.9768884
xlarge	wrack	10	0.9630782	0.0110165	0.9342632	0.9795391
xlarge	wrack	11	0.9662152	0.0104701	0.9384699	0.9816936
xlarge	wrack	12	0.9688534	0.0099892	0.9420565	0.9834751
xlarge	wrack	13	0.9711035	0.0095612	0.9451579	0.9849695
xlarge	wrack	14	0.9730456	0.0091768	0.9478715	0.986239
xlarge	upper	1	0.7472436	0.0566377	0.621568	0.8418051
xlarge	upper	2	0.8505682	0.0307174	0.779951	0.9013884
xlarge	upper	3	0.8930659	0.022112	0.8413927	0.9293185
xlarge	upper	4	0.9163929	0.0182008	0.8731063	0.9458295
xlarge	upper	5	0.9311855	0.0160001	0.8924147	0.9566629

Model-averaged estimates (cont.)

Carcass Size	Position on Beach	Day	Estimate	Standard Error	Lower Confidence Interval	Upper Confidence Interval
xlarge	upper	6	0.9414253	0.0145607	0.9054716	0.9642442
xlarge	upper	7	0.9489453	0.0135169	0.9149491	0.9698012
xlarge	upper	8	0.9547084	0.0127053	0.9221825	0.9740222
xlarge	upper	9	0.9592699	0.0120437	0.927911	0.977321
xlarge	upper	10	0.9629725	0.0114862	0.932578	0.9799593
xlarge	upper	11	0.9660397	0.0110051	0.9364656	0.9821106
xlarge	upper	12	0.9686234	0.0105827	0.9397624	0.9838936
xlarge	upper	13	0.9708304	0.0102068	0.9425997	0.9853918
xlarge	upper	14	0.972738	0.0098687	0.9450716	0.986666

MARSH EDGE CARCASS PERSISTENCE
Model-averaged estimates of daily persistence probability for bird carcasses on marsh edges in the northern Gulf of Mexico, for each combination of time, marsh type, and carcass size (NOTE: models that did not converged were not used in model averaging).

Marsh Type	Carcass Size	Day	Estimate	Standard Error	Lower Confidence Interval	Upper Confidence Interval
Phragmites	small	1	0.1515666	0.0889457	0.0440217	0.4093437
Phragmites	small	2	0.4123014	0.1536454	0.168363	0.7085504
Phragmites	small	3	0.6079354	0.2124701	0.2127307	0.8989695
Phragmites	small	4	0.7170829	0.216657	0.2381023	0.9536107
Phragmites	small	5	0.7788995	0.2051799	0.2542805	0.9732587
Phragmites	small	6	0.8171599	0.1919365	0.2648993	0.9822786
Phragmites	small	7	0.8429694	0.1797347	0.2727335	0.9871538
Phragmites	small	8	0.8616158	0.1689141	0.2793117	0.9901016
Phragmites	small	9	0.8758067	0.1593514	0.2853307	0.9920356
Phragmites	small	10	0.8870392	0.1508644	0.2910744	0.9933856
Phragmites	small	11	0.8962005	0.1432885	0.2966455	0.9943741
Phragmites	medium	1	0.3719941	0.0993155	0.2047417	0.5767816
Phragmites	medium	2	0.5476785	0.0878591	0.3766385	0.7081532
Phragmites	medium	3	0.6479522	0.086907	0.4658821	0.7952366
Phragmites	medium	4	0.7124225	0.087373	0.5178187	0.8510744
Phragmites	medium	5	0.7572354	0.0868201	0.5527786	0.8872806
Phragmites	medium	6	0.7901387	0.0853057	0.5787007	0.9116605
Phragmites	medium	7	0.8152968	0.0832012	0.5991404	0.9287549
Phragmites	medium	8	0.835142	0.0807961	0.6159303	0.941184
Phragmites	medium	9	0.8511879	0.0782748	0.6301248	0.9505061
Phragmites	medium	10	0.8644249	0.075747	0.6423825	0.9576842
Phragmites	medium	11	0.8755274	0.0732755	0.6531411	0.963336
Phragmites	large	1	0.4838128	0.1105218	0.2824843	0.6905365
Phragmites	large	2	0.6633053	0.0839874	0.4852464	0.8045768
Phragmites	large	3	0.7526481	0.0752037	0.5795766	0.8704041
Phragmites	large	4	0.8054709	0.0703504	0.6320066	0.9089479
Phragmites	large	5	0.8401655	0.0662786	0.6664311	0.9325686
Phragmites	large	6	0.8646162	0.0625347	0.6914858	0.9479092
Phragmites	large	7	0.8827377	0.059082	0.710928	0.9584067
Phragmites	large	8	0.8966851	0.0559216	0.7266698	0.9659096
Phragmites	large	9	0.9077394	0.0530433	0.7398037	0.9714666
Phragmites	large	10	0.9167087	0.0504266	0.7510081	0.9757051
Phragmites	large	11	0.924127	0.0480472	0.7607323	0.9790179
Phragmites	xlarge	1	0.5690979	0.1175561	0.3404224	0.7716683
Phragmites	xlarge	2	0.6790211	0.0739098	0.5211421	0.8043858
Phragmites	xlarge	3	0.7357513	0.0659359	0.5888683	0.8440549

Model-averaged estimates (cont.)

Marsh Type	Carcass Size	Day	Estimate	Standard Error	Lower Confidence Interval	Upper Confidence Interval
Phragmites	xlarge	4	0.7713426	0.0675103	0.6143606	0.8771956
Phragmites	xlarge	5	0.7961362	0.0706585	0.6245803	0.9016418
Phragmites	xlarge	6	0.8145901	0.0735376	0.6285024	0.9194157
Phragmites	xlarge	7	0.8289694	0.0758465	0.62944	0.9325708
Phragmites	xlarge	8	0.840557	0.077627	0.6288101	0.9425482
Phragmites	xlarge	9	0.8501382	0.0789797	0.6272982	0.9502978
Phragmites	xlarge	10	0.8582229	0.0799982	0.6252687	0.9564468
Spartina	xlarge	11	0.865158	0.0807574	0.6229299	0.9614177
Spartina	small	1	0.2671057	0.1164729	0.1019712	0.5391194
Spartina	small	2	0.585866	0.1273454	0.3358539	0.798288
Spartina	small	3	0.7505141	0.148281	0.3891695	0.9342274
Spartina	small	4	0.8272654	0.1392813	0.4148303	0.9700196
Spartina	small	5	0.8681015	0.1261364	0.4317068	0.9827653
Spartina	small	6	0.8928508	0.1142825	0.4449335	0.9885874
Spartina	small	7	0.9093852	0.1042682	0.4566337	0.991725
Spartina	small	8	0.921235	0.0958481	0.4676015	0.9936205
Spartina	small	9	0.9301742	0.0887114	0.4780952	0.9948644
Spartina	small	10	0.9371806	0.0825991	0.4881844	0.9957327
Spartina	small	11	0.9428357	0.0773098	0.4978786	0.9963683
Spartina	medium	1	0.5537745	0.0805889	0.3957327	0.7016451
Spartina	medium	2	0.7119151	0.0526226	0.5991214	0.8033873
Spartina	medium	3	0.7871148	0.0491999	0.6752731	0.8679679
Spartina	medium	4	0.831101	0.0487371	0.7136014	0.9066974
Spartina	medium	5	0.8599792	0.0479564	0.7377928	0.9305847
Spartina	medium	6	0.8803976	0.0467555	0.7550813	0.9461654
Spartina	medium	7	0.8956015	0.045327	0.7683655	0.9568703
Spartina	medium	8	0.9073639	0.0438145	0.7790588	0.96455
Spartina	medium	9	0.9167355	0.0423013	0.7879489	0.9702576
Spartina	medium	10	0.9243787	0.0408315	0.7955175	0.9746243
Spartina	medium	11	0.9307318	0.039427	0.8020787	0.9780465
Spartina	large	1	0.6631652	0.0760695	0.5024918	0.7932939
Spartina	large	2	0.8012979	0.0423217	0.7054618	0.8716255
Spartina	large	3	0.8598348	0.0351169	0.776053	0.9156783
Spartina	large	4	0.8920145	0.0323531	0.8104856	0.941022
Spartina	large	5	0.9123148	0.0303971	0.8316646	0.956353
Spartina	large	6	0.9262671	0.0286916	0.8464869	0.9662395
Spartina	large	7	0.9364359	0.027146	0.8576817	0.972983
Spartina	large	8	0.9441708	0.0257402	0.8665638	0.9777978
Spartina	large	9	0.9502489	0.0244628	0.8738564	0.9813647
Spartina	large	10	0.9551488	0.0233021	0.8799965	0.9840879

Model-averaged estimates (cont.)

Marsh Type	Carcass Size	Day	Estimate	Standard Error	Lower Confidence Interval	Upper Confidence Interval
Spartina	large	11	0.9591815	0.0222462	0.8852671	0.9862193
Spartina	xlarge	1	0.733377	0.0874312	0.5338068	0.8685518
Spartina	xlarge	2	0.8119167	0.045572	0.7063243	0.8856873
Spartina	xlarge	3	0.8479377	0.0386286	0.7560835	0.9093487
Spartina	xlarge	4	0.8693003	0.0396437	0.7704577	0.9294765
Spartina	xlarge	5	0.8837131	0.0417881	0.7740033	0.9440166
Spartina	xlarge	6	0.8942275	0.0437123	0.7736184	0.9543698
Spartina	xlarge	7	0.9023115	0.0452413	0.7715689	0.961917
Spartina	xlarge	8	0.9087658	0.0464221	0.7687458	0.9675816
Spartina	xlarge	9	0.9140673	0.0473285	0.7655514	0.9719501
Spartina	xlarge	10	0.9185194	0.0480245	0.7621858	0.9753999
Spartina	xlarge	11	0.922325	0.0485589	0.7587557	0.9781798
